# Supplementary material for: A Search for Anti-Naegleria fowleri Agents Based on Competitive Exclusion Behavior of Microorganisms in Natural Aquatic Environments
Source: Pathogens. 2021 Feb 1;10(2):142. doi: 10.3390/pathogens10020142 (PMC7911858; doi:10.3390/pathogens10020142)
Supplement: Supplementary file 1 [file pathogens-10-00142-s001.pdf]

pathogens

MDPI

Supplementary data

A search for anti-*Naegleria fowleri* agents based on competitive exclusion behavior of microorganisms in natural aquatic environments

Pichet Ruenchit [1,*], Narisara Whangviboonkij [1], Hathai Sawasdipokin [1], Uraporn Phumisantiphong [2] and Wanpen Chaicumpa [3]

[1] Department of Parasitology, Faculty of Medicine Siriraj Hospital, Mahidol University, Bangkok 10700, Thailand; narisara.wha@mahidol.ac.th (N.W.); hathai.noc@mahidol.ac.th (H.S.)
[2] Department of Clinical Pathology, Faculty of Medicine Vajira Hospital, Navamindradhiraj University, Bangkok 10300, Thailand; uraporn@nmu.ac.th
[3] Center of Research Excellence on Therapeutic Proteins and Antibody Engineering, Department of Parasitology, Faculty of Medicine Siriraj Hospital, Mahidol University, Bangkok 10700, Thailand; wanpen.cha@mahidol.ac.th
* Correspondence: pichet.rue@mahidol.edu; Tel.: +662 419 6484; Fax: +662 419 6470

Citation: Ruenchit, P.; Whangviboonkij, N.; Sawasdipokin, H.; Phumisantiphong, U.; Chaicumpa, W. A Search for Anti-*Naegleria fowleri* Agents Based on Competitive Exclusion Behavior of Microorganisms in Natural Aquatic Environments. *Pathogens* **2021**, *10*, 142. https://doi.org/10.3390/pathogens10020142

Received: 22 December 2020
Accepted: 28 January 2021
Published: 1 February 2021

Publisher's Note: MDPI stays neutral with regard to jurisdictional claims in published maps and institutional affiliations.

Copyright: © 2021 by the authors. Submitted for possible open access publication under the terms and conditions of the Creative Commons Attribution (CC BY) license (http://creativecommons.org/licenses/by/4.0/).

Figure S1. Attachment to the flask plastic surface of the remained viable trophozoites and the floating trophozoites after CFSs removal.

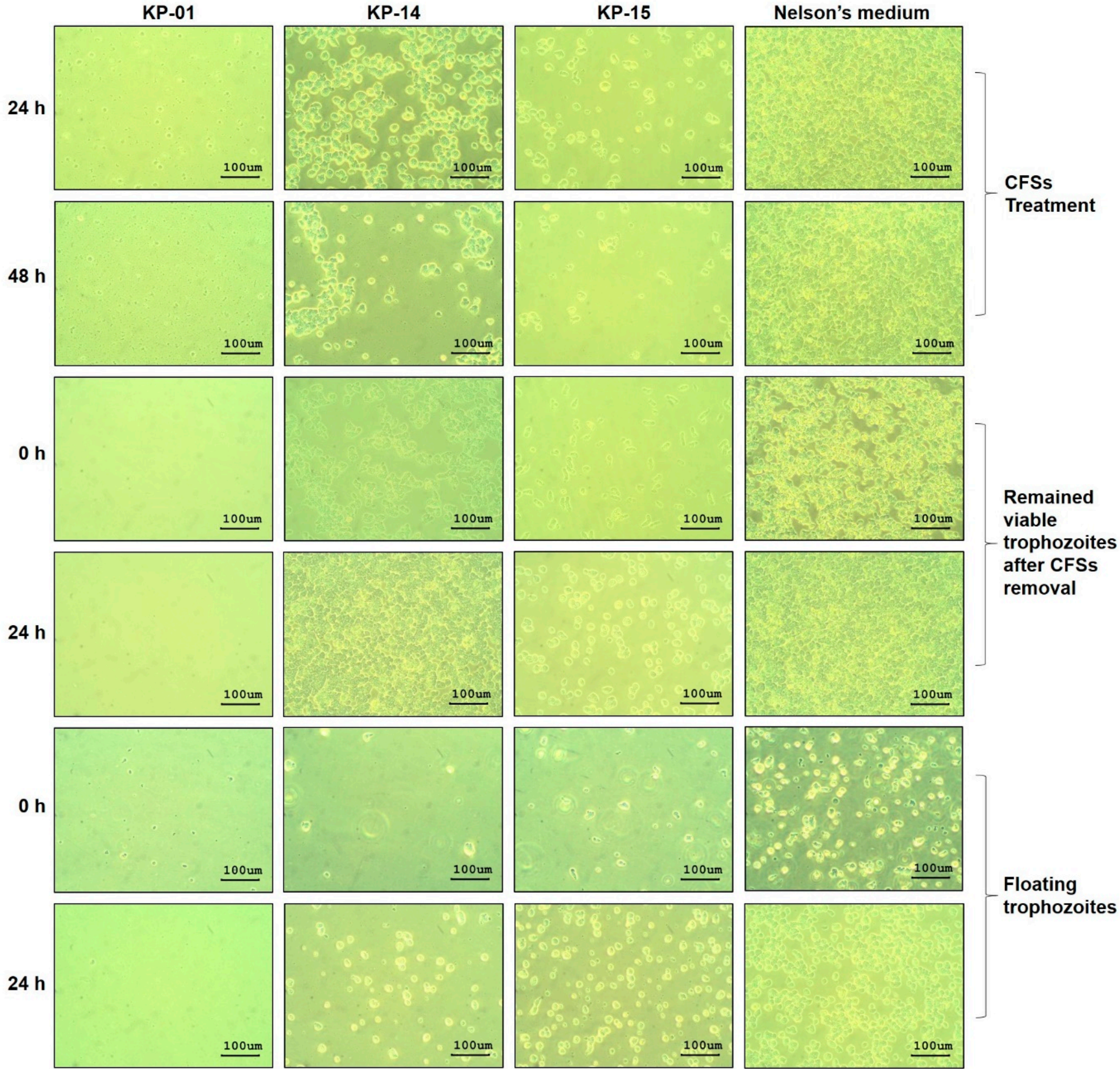

Figure S1. Attachment to the flask plastic surface of the remained viable trophozoites and the floating trophozoites after CFSs removal. *N. fowleri* (5×10^5 cells) in a T 25-cm^2 flask were treated with 1.60 mg mL^{-1} of CFSs of KP-01, KP-14, and KP-15, and Nelson's medium alone. CFSs of the KP-01, KP-14, and KP-15 were removed after 48 h-post-treatment and fresh medium was replaced. In the meantime, the floating trophozoites were collected from the removed medium containing CFSs by centrifugation, resuspended in fresh medium, and transferred to the new T 25-cm^2 flask. Attachment of the remained viable trophozoites and the floating trophozoites was observed and recorded under the Olympus IX70 Inverted Tissue Culture Microscope (Olympus, Tokyo, Japan) at 200× magnification. Scale bars representing 100 µm in size were set using Micro Capture Version 7.9.
